# Vector‐Borne Hemopathogens in Goats: Molecular Identification and Genetic Diversity in Sulaymaniyah, Iraq

**DOI:** 10.1155/vmi/3750343

**Published:** 2026-08-07

**Authors:** Shadan Hassan Abdullah

**Affiliations:** ^1^ Microbiology Department, College of Veterinary Medicine, University of Sulaimani, Sulaymaniyah 46001, Iraq, univsul.edu.iq

**Keywords:** *Anaplasma*, goat, PCR, phylogeny, Sulaymaniyah, *Theileria*

## Abstract

Tick‐borne diseases impose significant constraints on livestock, caused by various pathogens that affect health and reduce reproductive performance. The study aimed to determine the occurrence rates of hemopathogens, coinfection, and genetic diversity in native goats from four regions in Sulaymaniyah Province, Iraq. Blood samples were randomly collected from 250 apparently clinically healthy goats. Conventional PCR was conducted targeting the 18S rRNA and MSP‐4 genes to detect *Theileria* spp. and *Anaplasma ovis*, respectively, and to analyze genetic diversity. The overall infection rate of hemopathogens in goats was 67.6%, with a higher rate of *Theileria* spp. (49.2%) compared to *A. ovis* (38.8%). Coexistence of both pathogens was also observed in 20.4% of the examined goats. The presence of *T. ovis* was verified by BLAST analysis of 18S rRNA sequence isolates. Phylogenetic analysis revealed that recent isolates PV575344.1 and PV575345.1 were grouped along with *T. ovis* isolates from Iraq, Iran, Türkiye, and India. Sequence analysis of the *Anaplasma ovis* msp4 gene revealed that the study isolates PV222122.1 and PV222123.1 were highly similar to previous isolates in GenBank and clustered with them in the constructed phylogenetic tree. The current findings revealed the presence of vector‐borne hemopathogens and the cocirculation of *T. ovis* and *A. ovis* with a higher infection rate in asymptomatic goats, indicating their endemicity. Attention to animal health is essential to reduce financial losses, and further study on tick vector inspection and the zoonotic potential of *Anaplasma* spp. is essential.

## 1. Introduction

Vector‐borne diseases account for a significant proportion of infectious diseases, which are caused by a multitude of pathogens [1]. Food security depends heavily on small ruminants, which contribute significantly to milk and meat production. They are prone to a variety of health problems, including infectious and parasitic disorders, which are known or reported to lower their productivity significantly [2]. Various tick species are primary vectors of several pathogens, including *Theileria, Babesia, Anaplasma*, and *Hepatozoon* [3].

The impact of TBDs has been linked to significant economic losses due to mortalities, higher expenses for managing herd health, and reduced production of meat, milk, and wool [4]. Although common in small ruminants, TBDs are frequently overlooked in endemic countries due to robust tolerance, accompanied by acquired natural immunity in infected animals and the absence of severe clinical signs during infection [5, 6]. The risk of TBDs has escalated due to the expansion of tick vectors driven by environmental and climatic changes [7]. Livestock are particularly vulnerable to various TBDs, which may lead to serious outcomes like anemia, emaciation, decreased weight and productivity, abortion, and even death [8].

Theileriosis is a prevalent hemoprotozoan infection transmitted by ticks, caused by species of the genus *Theileria*, and affects both domestic and wild animals globally [9]. Various *Theileria* spp. affect sheep and goats, while some *Theileria* species, like *T. ovis* (etiology of benign theileriosis) and *T. recondita* (cause mild ovine theileriosis), infrequently or never cause disease. However, other *Theileria* spp. that affect small ruminants, such as *T. lestoquardi* (causes malignant ovine theileriosis), *T. uilenbergi,* and *T. luwenshuni,* are thought to be highly pathogenic [10].

Anaplasmosis is a highly prevalent tick‐borne infection with global distribution, particularly in tropical and subtropical regions [11]. The genus *Anaplasma* comprises various species that cause disease in a wide range of hosts and are transmitted by ticks; some are zoonotic, causing anaplasmosis in humans [3]. In small ruminants, it is caused by *A. ovis, A. phagocytophilum, A. capra,* and *A. marginale* [6]. Compared with other species, anaplasmosis in small ruminants is most commonly caused by *Anaplasma ovis* and has fewer harmful consequences [12]. The infection is usually asymptomatic, but rickettsiaemia can occasionally lead to fever, severe anemia, weight loss, decreased milk production, abortion, or even death in goats during acute infections. Apart from *A. phagocytophilum,* which is a known zoonotic pathogen, there is also evidence of the zoonotic potential of *A. ovis* and *A. capra* [11].

Hemopathogens, including *Theileria* and *Anaplasma,* are transmitted to vertebrate hosts by the bite of Ixodidae ticks such as *Rhipicephalus, Hyalomma*, *Dermacentor*, *Haemaphysalis*, *Ixodes,* and *Amblyomma* [13, 14]. Several economically significant characteristics, including short reproductive cycles, high reproduction rates, and high consumer acceptability, make goats a valuable food animal species with a special ability to adapt to stressful environments [15].

Due to their high sensitivity and specificity, polymerase chain reaction (PCR) assays are a useful technique for detecting and characterizing pathogen species in both animals and tick vectors, particularly when dealing with challenging circumstances such as low parasite burden or coinfections [16]. Vector ‐borne diseases caused by *T. ovis* and *A. ovis* have substantial financial implications. *Theileria* species are often identified using the 18S rRNA gene [17], while *Anaplasma* species are typically identified using the MSP4 gene [18].

Numerous investigations have been conducted in the study area regarding theileriosis and anaplasmosis in sheep and cattle. To address the knowledge gap regarding hemopathogen infection in goats, the current study was designed to determine the occurrence of *Theileria* and *Anaplasma* in native goats using conventional PCR. Additionally, the study aimed to evaluate their genetic diversity based on the 18S rRNA gene for *T. ovis* and the msp1*α* gene for *A. ovis*.

## 2. Materials and Methods

### 2.1. Study Design and Sampling Conditions

A total of 250 native goats were randomly selected from 10 small‐ruminant farms, whose owners consented to participate. No particular health issues were found in the animals chosen.

The local domestic breed of goat (*Capra hircus*) was sampled from small ruminant farms across four regions in Sulaymaniyah Province, northeast of Iraq, between (35°04′–36°30′) latitude and (44°50′–46°16′) longitude.

The goats ranged in age from six months to four years, irrespective of sex. This cross‐sectional study was conducted between April and October 2023. The animals were raised under a semi‐extensive rearing system, allowing goats to graze on open pastures, which increased the risk of tick infestation and exposure to tick‐borne pathogens.

### 2.2. Blood Sample Collection

Approximately 4 mL of blood was collected from the jugular vein in EDTA tubes using disposable syringes. Samples were transported to the laboratory at the Veterinary Medicine College, University of Sulaimani, using an isothermal container and stored at −80°C until DNA extraction.

### 2.3. DNA Extraction and PCR Amplification

Genomic DNA was extracted from 200µL of whole blood samples using the GeNet Bio DNA extraction kit (GeNet Bio, South Korea) according to the manufacturer’s instructions. The extracted DNA aliquots were then stored at −20°C for PCR analysis.

The DNA aliquots obtained were screened by PCR analysis using primer sets targeting 18S rRNA for *Theileria* spp. and the msp4 gene for *A. ovis. Theileria* spp. was identified using 450 bp amplification with previously produced primer sets of RLB‐F [5′‐GACACAGGGAGGT AGTGACAAG3′] and RLB‐R [5′‐CTAAGAATTTCACC TCTGACAGT‐3′] [19], and *A. ovis* was identified via amplification of 831 bp with forward [5′‐TTGTTTACA GGGGGCCTGTC‐3′] and reverse [5′‐GAACAGGAATCTTGCTCC AAG‐3′] primer sets [20].

The amplification was carried out in a 20 µL total reaction volume using an automated Prime Thermal cycler with different thermoprofiles. For *Theileria* spp., the cycling conditions involved an initial denaturation at 94°C for 2 min, followed by 35 cycles of 94°C for 30 s, 57°C for 30 s, and 72°C for 30 s, with a final extension at 72°C for 5 min. The reaction conditions for *A. ovis* were as follows: an initial denaturation at 94°C for 5 min, followed by 30 cycles at 94°C for 30 s, 55.5°C for 30 s, and 72°C for 45 s, and an extension step at 72°C for 5 min. PCR products were resolved loaded on a 1% agarose gel and visualized under UV light.

### 2.4. Sequencing and Phylogenetic Analysis

Of the total 169 positive samples, two PCR products were selected and sequenced for each pathogen in both directions using the same PCR amplification primers at a Macrogen sequencing facility (Macrogen Inc., Seoul, Korea) to confirm the pathogens.

The identified partial sequences were logged in NCBI (the National Center for Biotechnology Information: https://blast.ncbi.nlm.nih.gov/Blast.cgi) for sequence alignment and homology analysis using BLASTn after submission to GenBank. The representative sequences from this study were deposited in GenBank and assigned accession numbers PV575340.1 and PV575341.1 for *T. ovis* and PV222122.1 and PV222123.1 for *A. ovis.* The MEGA 10 software was used to build the phylogenetic trees using the neighbor‐joining algorithm [21]. Tree stability was estimated using a bootstrap analysis with 1000 replications [22], and the evolutionary distances were calculated using the p‐distance method [23]. Multiple sequence alignment was performed using Clustal W.

### 2.5. Data Analysis

The obtained study data were analyzed using SPSS V29.0. To evaluate the association between variables, including detected hemopathogens and the sampling sites, the chi‐square (*χ* ^2^) test was used, and significance was estimated at 99%; *p* values < 0.01 were considered statistically significant. A 95% confidence interval was evaluated for the sampling sites.

## 3. Results

The data revealed the presence of vector‐borne pathogens, with a high prevalence rate of 67.6%. The recovered hemopathogens, *Theileria ovis* and *Anaplasma ovis*, were detected at prevalence rates of 49.2% and 38.8%, respectively. Statistical analysis revealed no significant result at *p* < 0.01 (Table 1). Coinfection with both pathogens was common, observed in 51 goats (20.4%), as shown in Table 2.

**TABLE 1:** Frequency of *Theileria* and *Anaplasma* infection among goats in Sulaymaniyah Provinc**e**.

Pathogen	No. of +ve	% of +ve	Chi‐square value (*p* value)
*Theileria*	123	49.2	5.48
*Anaplasma*	97	38.8	0.019

*Note:* Not significant at *p* < 0.01.

**TABLE 2:** Pattern of hemopathogen infection among goat populations in Sulaymaniyah Province.

Infection status	Pathogen	No. of infected goats	Detection rate %
Single infection	*Theileria*	72	28.8
	*Anaplasma*	46	18.4

Coinfection		51	20.4

Total		169	67.6

The data in Table 3 showed the distribution of hemopathogens’ occurrence at the study sites, with no significant association (*p* < 0.01), despite variation in prevalence rates. The results in Table 4 showed the distribution of *Theileria* and *Anaplasma* positivity within the study sites. The highest *Theileria* infection rate was reported among the examined goats from the Sharazoor region (56.9%), and the lowest rate was in Bazian (40.0%). Concerning *Anaplasma,* the highest infection rate was reported in Tanjaro (45.0%), and the lowest was reported in Sitak (33.3%); however, statistical analysis showed no significant association at *p* < 0.01.

**TABLE 3:** Frequency of hemopathogen infection among the goat population in Sulaymaniyah Province according to the study sites.

Locations	Total examined no.	No. of positive goats	Frequency of infection	Confidence interval 95%	Chi‐square value [*p* value]
Bazian	65	43	66.15	[54.65–77.65]	0.70 [0.8]
Sharazoor	65	44	67.69	[56.32–79.06]	
Said Sadiq	60	43	71.67	[60.26–83.06]	
Sitak	60	39	65.00	[52.93–77.06]	
Total	250	169	67.60		

*Note:* Not significant at *p* < 0.01.

**TABLE 4:** Distribution of *Theileria* and *Anaplasma* infection among the goat population in Sulaymaniyah Province according to the study sites.

Locations	Total no.	*Theileria* no. of positive	Frequency of infection	*p* value	*Anaplasma* no. of positive	Frequency of infection	*p* value
Bazian	65	26	40.0		28	43.1	0.4
Sharazoor	65	37	56.9	0.2	22	33.9	
Said Sadiq	60	29	48.3		27	45.0	
Sitak	60	31	51.7		20	33.3	
Total	250	123			97		

*Note:* Not significant at *p* < 0.01.

### 3.1. Phylogenetic Analysis

**FIGURE 1:**
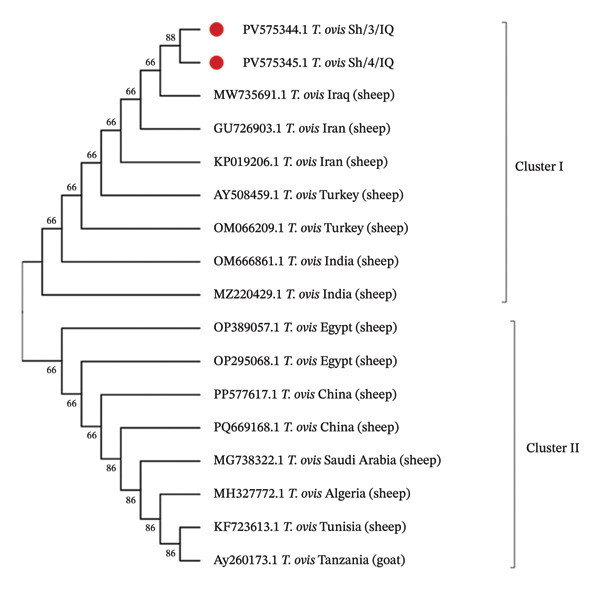
Phylogenetic relationships of *Theileria ovis* sequence isolates of goats in Sulaymaniyah Province with previously reported *T. ovis* isolates in the GenBank database were established. The tree was constructed based on 18S rRNA gene sequences using the neighbor‐joining method with the Kimura 3‐parameter model in MEGA X. Branching reliability was assessed by bootstrap analysis with 100 replicates.

The amplified DNA sequences of the partial 18S rRNA gene recovered in the current study were analyzed by BLAST, representing a high identity of 99% with *Theileria ovis* sequence isolates already available in the GenBank database. The topology of the *Theileria* sequencing isolates in the constructed phylogenetic tree indicated close links between *T. ovis* isolates, and the study isolates PV575344.1 and PV575345.1 were clustered with additional*T. ovis* isolates MW735691.1 from Iraq, GU726903.1 and KP019206.1 from Iran, AY508459.1 and OM066209.1 from Türkiye, OM666861.1 and MZ220429.1 from India (Figure 1).

**FIGURE 2:**
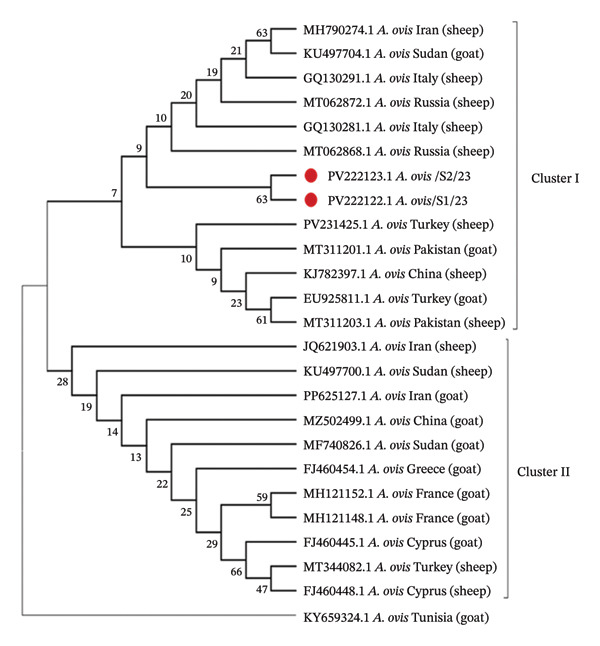
Phylogenetic relationships of *Anaplasma ovis* sequence isolates of goats in Sulaymaniyah Province with previously reported *A. ovis* isolates in the GenBank database were established. The tree was constructed based on MSP4 gene sequences using the neighbor‐joining approach with the Kimura 3‐parameter model in MEGA X. Branching reliability was assessed by bootstrap analysis with 100 replicates.

A sequence homology analysis of the msp4 gene from the current study *A. ovis* isolates showed 99% similarity with *A. ovis* sequences already available in GenBank, as observed in BLAST analysis. Phylogenetic analysis showed that the study isolates PV222122 and PV222123 were grouped together within the same cluster with *A. ovis* from Iran MH790274.1, PV231425.1, and EU925811.1 from Turkey, GQ130291.1 and GQ130281.1 from Italy, MT062872.1 and MT062868.1 from Russia, MT311201.1 and MT311203.1 from Pakistan, KJ782397.1 from China, and KU497704.1 (Figure 2).

**FIGURE 3:**
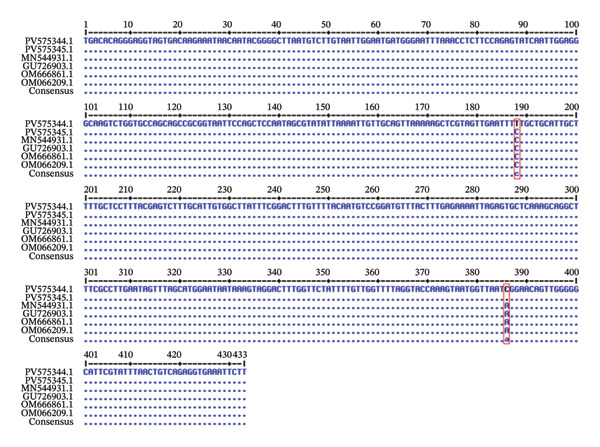
Sequence alignment analysis of *Theileria ovis* based on the 18S rRNA gene from goats in Sulaymaniyah Province with reference *T. ovis* sequences MN544931.1, GU726903.1, OM666861.1, and OM066209.1 in the GenBank database. Dots in the sequence signify the similarity.

**FIGURE 4:**
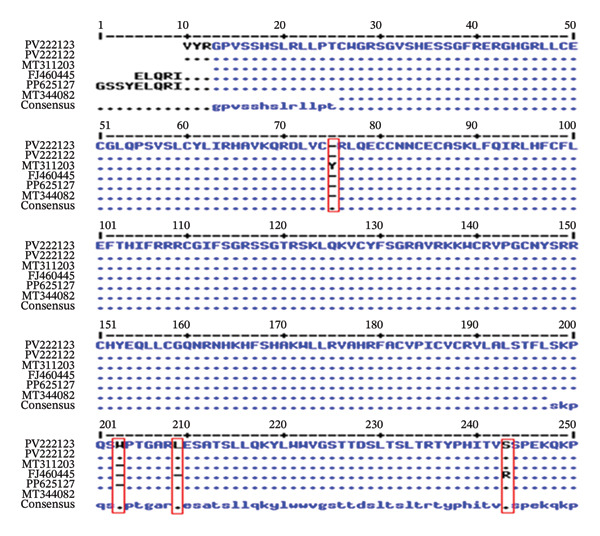
Sequence alignment analysis of *Anaplasma ovis* based on the msp4 gene from goats in Sulaymaniyah Province with reference *A. ovis* sequences MT311203.1, FJ460445.1, PP625127.1, and MT344082.1 in the GenBank database. Dots in the sequence signify the similarity.

Nucleotide sequences corresponding to *T. ovis* and *A. ovis* from the present work were subjected to multiple alignments. At the nucleotide level, the sequenced isolates of *T. ovis* PV575344.1 and PV575345.1 and *A. ovis* PV222122 and PV222123 showed more identity with isolates from other countries previously documented in the GenBank database (Figures 3 and 4).

## 4. Discussion

Goats are among the most significant farm animals for providing food for humans. In the current study, hemopathogens were detected by PCR, with a high infection rate of 67.6% which exceeds the reported data in Iran 42.4% and 38% [24, 25], 34.7% in Saudi Arabia [26], 32% in India [27], 30%, 14.3% in Pakistan [28, 29], 9.1% in Saudi Arabia [30] and 8.50% in Bangladesh [9], suggesting a higher disease burden in study regions. However, it remains lower than the exceptionally high prevalence of 85.3% reported in Iran [16] and 51.9% in Saudi Arabia [31].

The reported occurrence rate of anaplasmosis was 38.8%, which is consistent with the *Anaplasma* infection rate of 38.64% in the Philippines [6] and 39.3% in Pakistan [32]. Whereas it was higher than what was reported in China, 30.1% [11], in Egypt 21.3% [33], and 15.75% in Bangladesh [34], while in equivalent it was lower than the anaplasmosis infection rate of 78.91%, 71.2% in Türkiye [35, 36], 70.3% in Tunisia [37] and 52% in France [12].

Numerous factors might be associated with variation in reported prevalence rates that relate to the climate of the region, environment, tick population density, host susceptibility, and management systems [5, 38]. Frequent environmental exposure increases the risk of infections and makes them reservoirs for a variety of infectious agents, such as bacteria and protozoa [39]. Moreover, the extensive system of raising animals for pasture grazing directly affects diseases carried by ticks through increasing the possibility of exposure to natural vectors [34].

Dumanli et al. [40] stated that tick activity is generally highest between spring and autumn, which contributed to the sampling period in the current study. Velusamy et al. [41] contended that there is no seasonal impact and that variations are due to changes in the macroclimate, which is essential for tick reproduction. Other variables, such as host age, sample size, and detection techniques, also influence the frequency of tick‐borne pathogens [3].

Since *Rhipicephalus bursa* is the vector of numerous significant tick‐borne pathogens, the potential incidence of coinfections in goats warrants particular consideration [42, 43]. Coinfection and age‐dependent immunity are significant factors in tick‐borne diseases in small ruminants [44]. Anaplasmosis can also be transmitted mechanically through the biting of some hematophagous insects [34], such as tabanid flies [45], and *Melophagus ovinus* [46].

Coinfection with hemopathogens is common among small ruminants. In the current study, 20.4% of examined goats harbored both *Theileria* and *Anaplasma*, with higher infection rates of 28.8% for *Theileria* than 18.4% for *Anaplasma*. In agreement with current findings, Tumwebaze et al. [47] found that the infection rate of *Theileria* spp. (13.4%) was higher than that of *A. ovis* (5.5%) in Uganda. However, contrary results were reported, with a higher infection rate of *Anaplasma* (25.68%) than *Theileria* (10.81%) in India [44]. In addition, *T. ovis* had an infection rate of 14.3% in Pakistan, which was lower than that of *A. ovis* (16.6%) [28].

The data in Tables 3 and 4 show hemopathogen positivity at the sampling sites; theileriosis and anaplasmosis have also been observed, with differences in occurrence rates. Numerous factors related to environmental conditions, tick vectors, and the infected animal host might be associated. It was observed that infection rates increased with host age, resulting in an extension of the tick contact duration [11, 48]. Persistently infected hosts are crucial to the epidemiology of the diseases because they may act as tick reservoirs [49].

*Theileria ovis* is the most prevalent and the primary cause of theileriosis in small ruminants, resulting in significant economic losses in tropical and subtropical areas [50]. Despite not being as dangerous as *T. lestoquardi*, *T. ovis* causes an illness that can induce fever, progressive weight loss, decreased productivity, and even death [47]. Even though *A. ovis* is the most frequent cause of anaplasmosis in small ruminants, it is widely distributed and appears to be less pathogenic than other *Anaplasma* species, causing only subclinical infections with low‐grade fever [12].

Clinical indications related to TBDs were absent despite the high positive rate. This might reveal the endemicity of various infections in goats within the study area. According to Stuen [10], this could also be related to persistent infections, which are a feature of natural infections in areas where the disease is considered endemic. Because the pathogenicity of various species and genotypes varies depending on the host, the impact of subclinical infections should not be disregarded [51]. Furthermore, persistently infected individuals may serve as tick reservoirs, and these hosts are crucial in epidemiology [49]. Subclinical infection and innate resistance gained by *A. ovis* strains within endemic regions may make it challenging to determine the actual financial impact of *A. ovis* infection [52].

The 18S rRNA gene partial sequences derived from the current study showed remarkable conservation across the *Theileria* isolates; they were also highly identical to previous *Theileria ovis* isolates: MW735691.1 from Iraq, GU726903.1 and KP019206.1 from Iran, AY508459.1 and OM066209.1 from Türkiye, and OM666861.1 and MZ220429.1 from India. Genetic diversity of *A. ovis* has been stated, and the current isolates showed a high degree of similarity to previous isolates from Iran (MH790274.1, PV231425.1, and EU925811.1 in Turkey), Italy (GQ130291.1 and GQ130281.1), Russia (MT062872.1 and MT062868.1), Pakistan (MT311201.1 and MT311203.1), China, and Sudan (KJ782397.1).

## 5. Conclusion

A cross‐sectional study was conducted to determine the occurrence of hemopathogens among the goat population. The study’s findings represented a significant infection by vector‐borne hemopathogens. *T. ovis* and *A. ovis* are commonly identified species from clinically healthy goats, demonstrating the endemicity of the pathogens. A considerable rise in the number of imported small ruminants from nearby areas may make it more likely that hemopathogens may spread. *T. ovis* appears to be the main cause of theileriosis, and *A*. *ovis* was confirmed as the main etiology of anaplasmosis in goats in the study areas. Large‐scale investigations are required to find out the tick vector species transmitting *T. ovis* and *A. ovis*. Further investigations evaluating the risk factors for disease manifestation associated with tick‐borne pathogens are essential for improving livestock production and disease control.

## Funding

This research did not receive any specific grant.

## Ethics Statement

This study was reviewed and approved by the Ethical Committee of the Veterinary Medicine College, University of Sulaimani. Informed consent was obtained from the owners for taking blood samples from their animals.

## Conflicts of Interest

The author declares no conflicts of interest.

## Data Availability

The data that support the findings of this study are available on request from the corresponding author.
